# Supplementary material for: Strong homeostatic TCR signals induce formation of self‐tolerant virtual memory CD8 T cells
Source: EMBO J. 2018 May 11;37(14):e98518. doi: 10.15252/embj.201798518 (PMC6043851; doi:10.15252/embj.201798518)
Supplement: Supplementary file 1 — Expanded View Figures PDF [file EMBJ-37-e98518-s001.pdf]

# Expanded View Figures

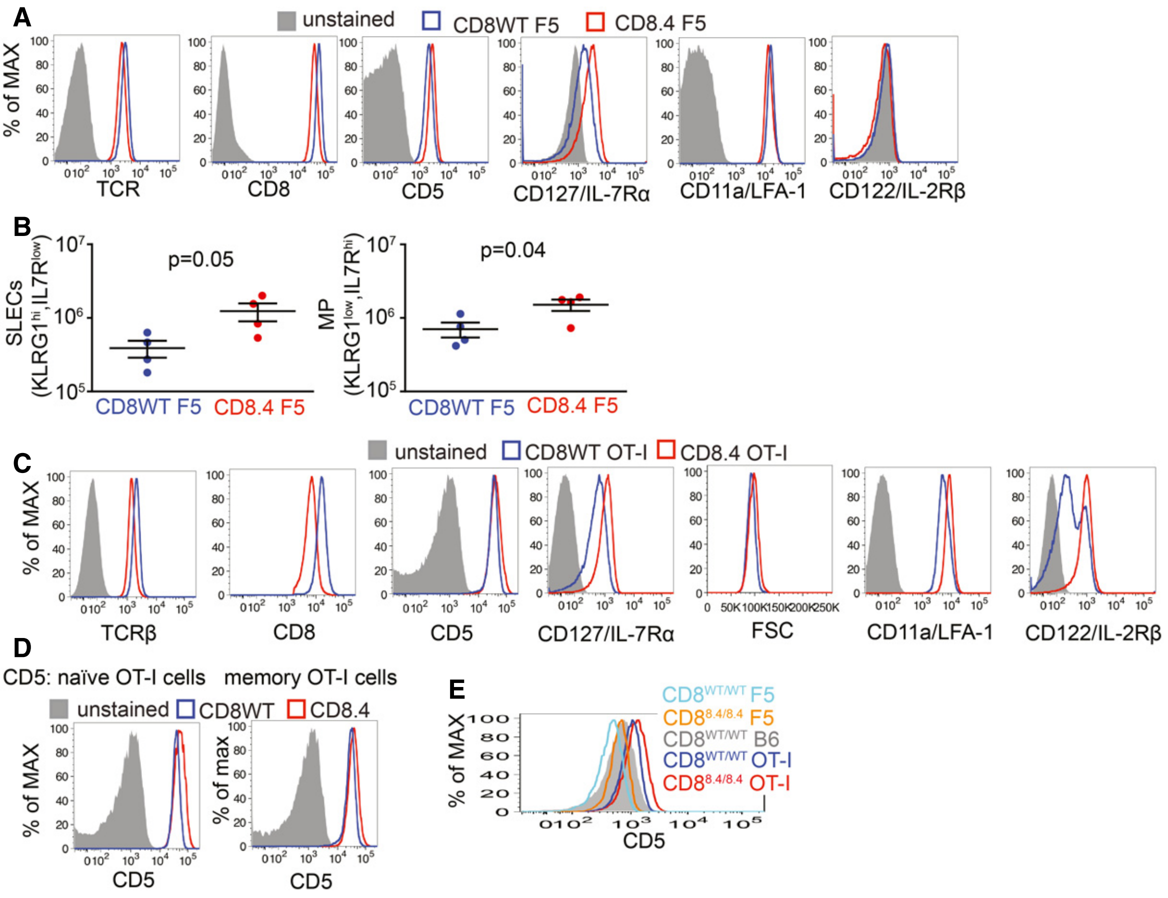


**Figure EV1. Analysis of CD8WT and CD8.4 monoclonal T cells, related to Fig 1.**

A Expression of indicated surface markers on CD8WT F5 and CD8.4 F5 LN T cell was analyzed by flow cytometry. A representative experiment out of four in total.

B CD8WT F5 and CD8.4 F5 T cells primed by Lm-NP68 (Fig 1D) were examined by flow cytometry. Absolute numbers of KLRG1$^{+}$ IL-7R$^{-}$ short-lived effector cells and KLRG1$^{-}$ IL-7R$^{+}$ memory precursors were determined. Mean ± SEM. $n$ = 4 mice per group from two independent experiments. Statistical significance was determined by two-tailed $t$-test. The data do not seem to be strongly deviated from the normal distribution and seem to have equal variance. However, because of the low $n$, we could not test the normality/equal variance rigorously.

C Expression of indicated markers on CD8WT OT-I and CD8.4 OT-I LN T cell and their size estimated by FSC signal was analyzed by flow cytometry. A representative experiment out of 10 in total.

D Expression of CD5 on CD44$^{-}$ (naïve) and CD44$^{+}$ (memory subsets) of CD8WT OT-I and CD8.4 OT-I LN T cells was analyzed by flow cytometry. A representative experiment out of three in total.

E Expression of CD5 on T cells isolated from LN of indicated mouse strains by flow cytometry. A representative experiment out of two in total.



© 2018 The Authors

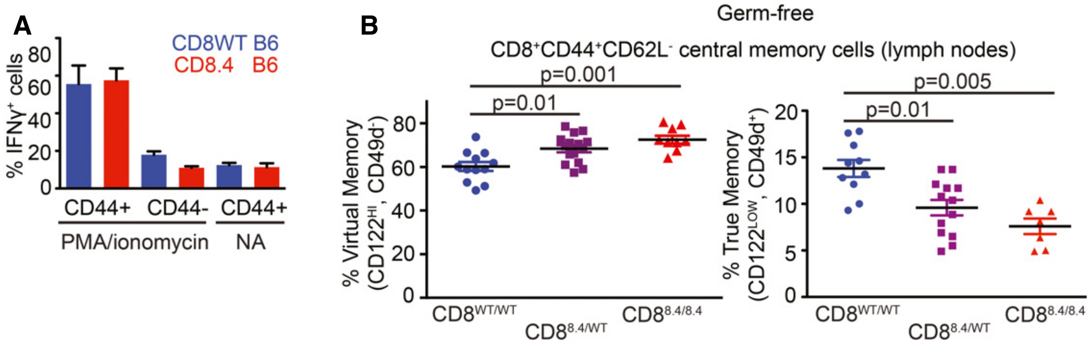


**Figure EV2. Analysis of CD8WT and CD8.4 polyclonal T cells, related to Fig 2.**

A LN cells isolated from polyclonal CD8WT and CD8.4 mice were stimulated with 10 ng/ml PMA and 1.5 μM ionomycin or left untreated for 5 h in the presence of BD Golgi Stop and the percentage of IFNγ-producing cells was determined by flow cytometry. The cells were gated as $CD8^+$ and further divided into $CD44^+$ and $CD44^-$ populations. Mean + SEM. $n$ = 4 independent experiments.

B Quantification of the frequency of $CD122^{HI}$ $CD49d^-$ VM and $CD122^{LOW}$ $CD49^+$ true memory cells among $CD8^+$ $CD44^+$ $CD62L^+$ CM T cells isolated from LN is shown. Mean, $n$ = 9–15 mice per group from four independent experiments. Statistical significance was determined using two-tailed Mann–Whitney test.

© 2018 The Authors

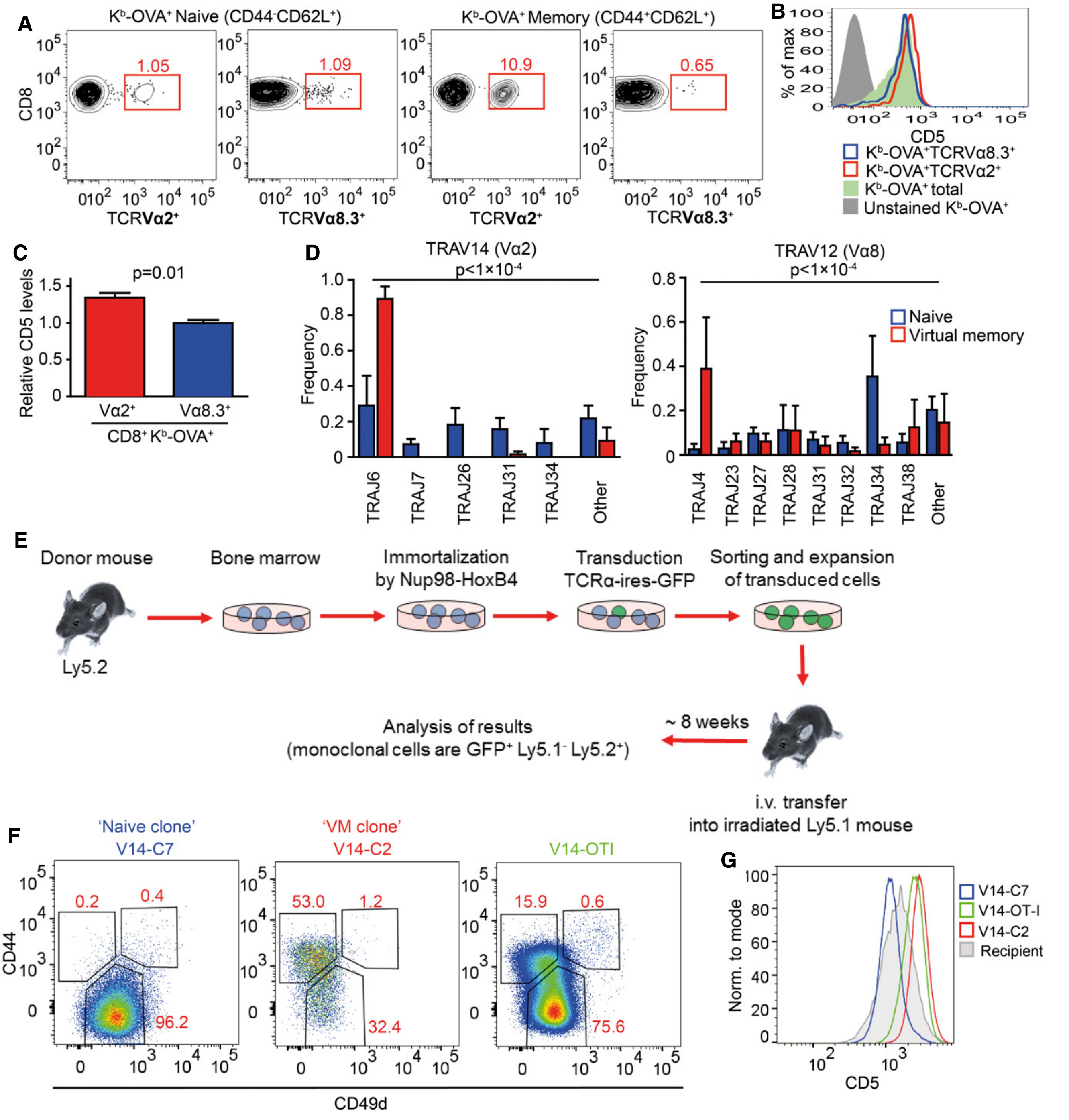


**Figure EV3. Differences in TCR repertoires between naïve and VM T cells, related to Fig 3.**

A Alternative gating strategy for the same samples as shown in Fig 3B–D (T cells from Vβ5 mice). A representative experiment out of eight in total.

B, C The expression of CD5 on $CD8^+$ $K^b$-OVA-$4mer^+$ $CD8^+$ $V\alpha2^+$ or $V\alpha8.3^+$ cells or total $CD8^+$ $K^b$-OVA-$4mer^+$ isolated from LN and the spleen was determined by flow cytometry. Relative MFI CD5 levels were quantified (CD5 levels on $CD8^+$ $K^b$-OVA-$4mer^+$ $V\alpha8.3^+$ were arbitrarily set as 1). Mean + SD, $n = 3$ independent experiments. Statistical significance was determined using two-tailed one-value *t*-test.

D TCRα sequences from Fig 3F were analyzed for TRAJ usage. Means + SEM. $n = 4$ independent experiments. Statistical significance was determined by chi-square test.

E Schematic representation of the generation and analysis of retrogenic monoclonal T-cell subsets.

F Frequency of VM T cells among monoclonal populations expressing TCR clones V14-C2, V14-C7, and OT-I generated as in Fig 3G.

G CD5 levels on naïve monoclonal T cells expressing V14-C2, V14-C7, and OT-I. Representative mice out of 9–14 in total from two to four independent experiments.


© 2018 The Authors

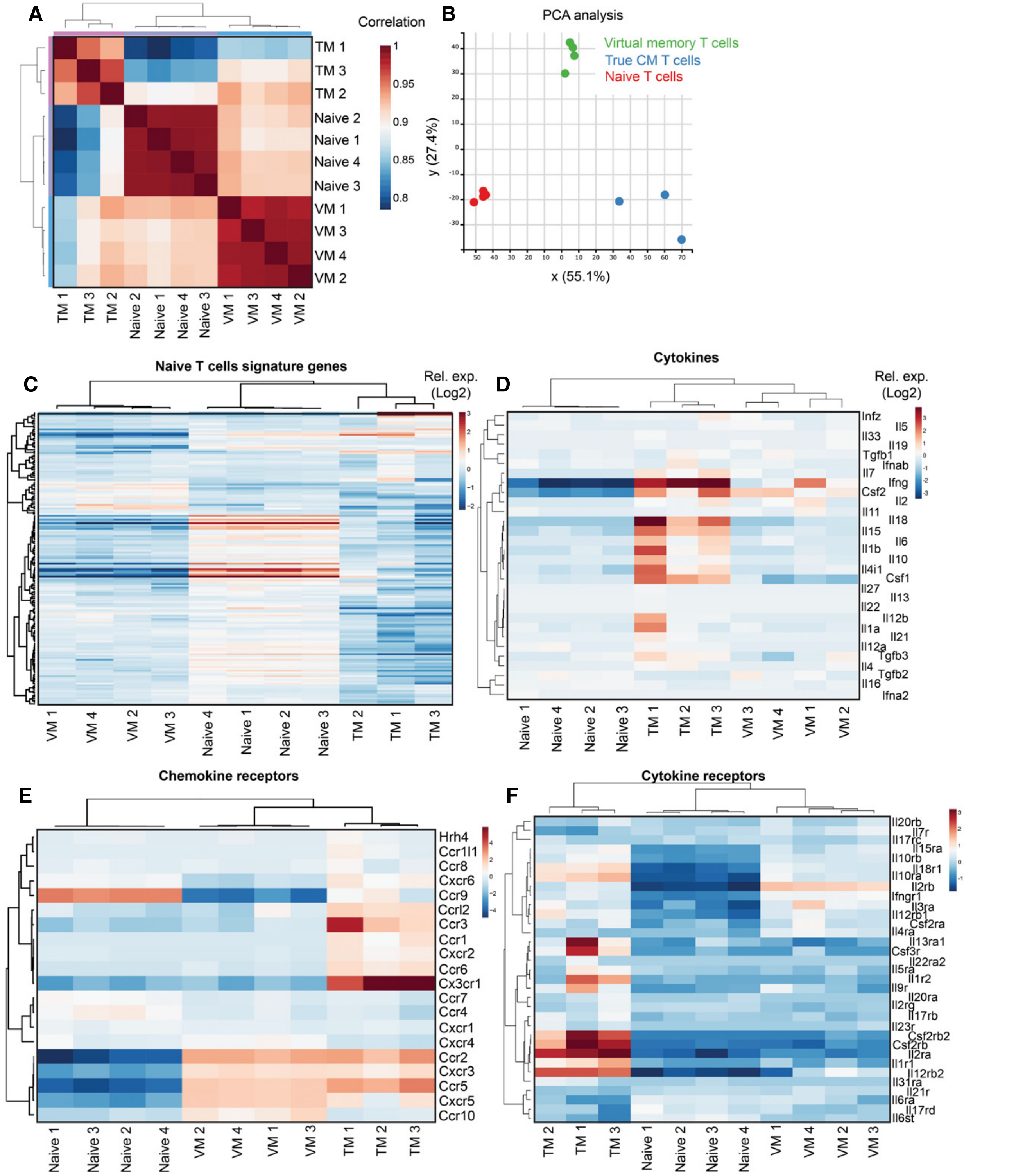


**Figure EV4. Gene expression analysis of naïve, VM, and TM T cells, related to Fig 4.**

Transcriptomes of naive ($n$ = 4), VM ($n$ = 4), and TM ($n$ = 3) $CD8^+$ T cells were analyzed by deep RNA sequencing.

A Correlation of the gene expression between individual samples.

B PCA analysis between the samples.

C–F Enrichment of $CD8^+$ naïve signature genes (as revealed by previous studies) (C), cytokine encoding genes (D), chemokine receptor encoding genes (E), and cytokine receptor encoding genes (F) in naïve, VM, and true CM T cells.

© 2018 The Authors

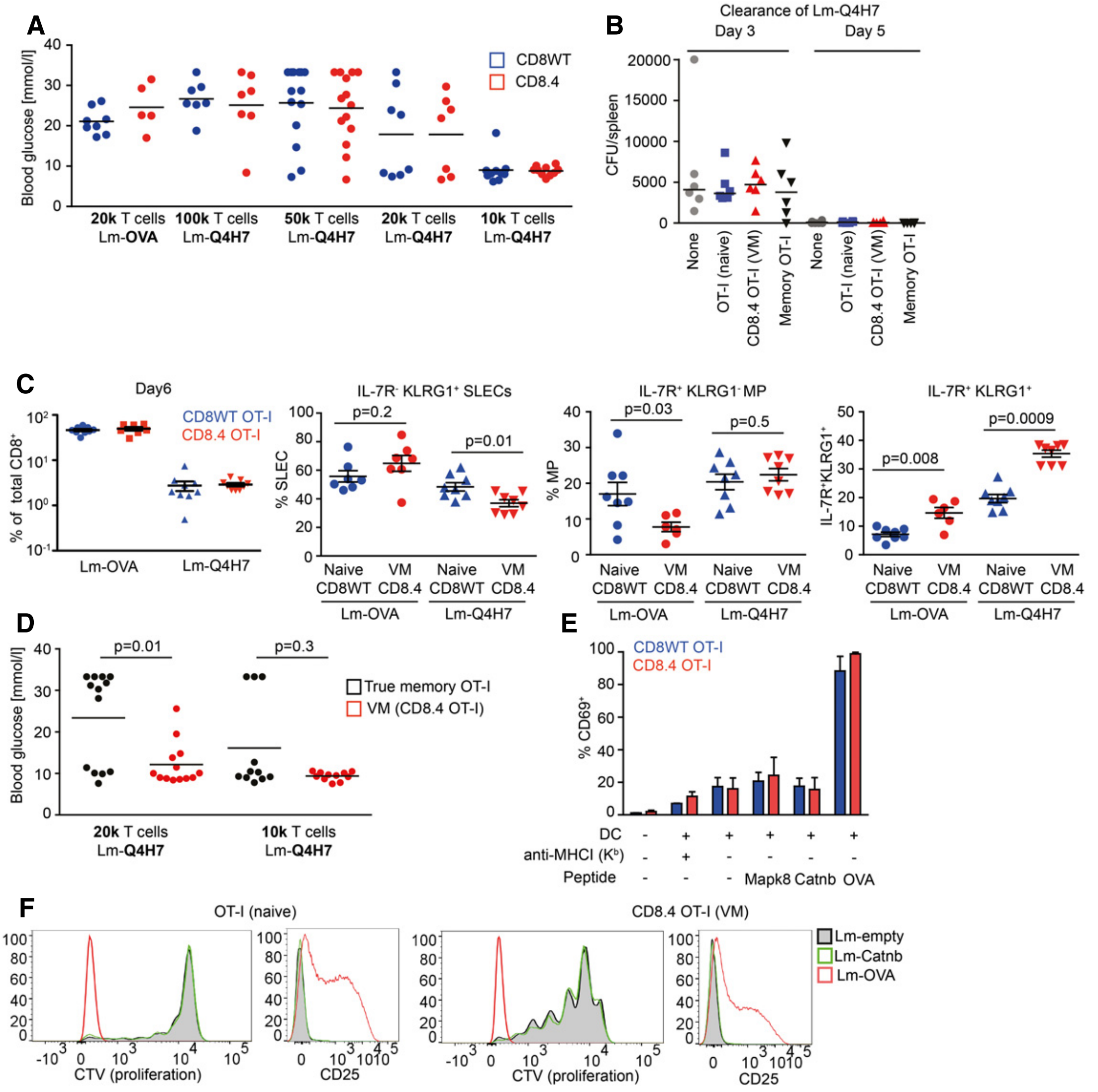

**Figure EV5. Functional characterization of naïve, VM, and TM T cells, related to Fig 5.**

A Indicated number of CD8WT OT-I or CD8.4 OT-I T cells were adoptively transferred into RIP-OVA hosts, which were infected with Lm-OVA or -Q4H7 1 day later. Blood glucose was measured 7 days after the infection. $n$ = 5–11 mice per group in 3–6 independent experiments. These are the same experiments as in Fig 5A. Mean is indicated.

B FACS-sorted 1 × $10^4$ naïve CD8WT OT-I, CD8.4 OT-I (VM), sorted true memory OT-I T cells or no T cells were adoptively transferred into Ly5.1 C57Bl/6 mice followed by infection with 5,000 CFU Lm-Q4H7. Total Lm-Q4H7 CFU counts in the spleen were quantified on day 3 and day 5 post-infection. $n$ = 6 mice in two independent experiments.

C Quantification of the experiment shown in Fig 5E and F. Percentage of donor OT-I and CD8.4 OT-I T cells among all $CD8^+$ T cells is shown. Percentage of short-lived effector cells (IL-$7R^-$ $KLRG1^+$), memory precursors (IL-$7R^+$ $KLRG1^-$), and double-positive cells IL-$7R^+$ $KLRG1^+$ among the donor cells are shown. Statistical significance was calculated using Mann–Whitney test. $n$ = 7 (CD8.4 OT-I + Lm-OVA) or 9 (the other three experimental conditions) in four independent experiments.

D Indicated number of CD8.4 OT-I or true memory OT-I T cells were adoptively transferred into RIP-OVA hosts, which were infected with Lm-Q4H7 1 day later. The glucose in the blood was measured on day 7 post-infection. Statistical significance was calculated using Mann–Whitney test $n$ = 11 (1 × $10^4$ transferred cells) or 13 (2 × $10^4$ transferred cells) mice per group in four independent experiments. These are the same experiments as in Fig 5H.

E OT-I or CD8.4 OT-I was cocultured with bone marrow dendritic cells that were pre-loaded or not with 10 μM putative endogenous positive selecting peptides for OT-I T cells (Mapk8, AGYSFEKL; Cantb, RTYTYEKL) or with 0.1 μM OVA peptide. Blocking of the MHCI by adding anti-H2-$K^b$ antibody (clone Y3, 10 μg/ml) was used as a negative control. Percentage of $CD69^+$ T cells in the overnight stimulation was quantified by flow cytometry. Mean + SEM. $n$ = 2 (MHC-I blocking) or 3 (all the other experimental conditions) independent experiments.

F CellTrace violet-loaded OT-I or CD8.4 OT-I T cells were adoptively transferred into Ly5.1 C57Bl/6 donors followed by infection with Lm-OVA (1 × $10^4$ transferred cells), Lm-Catnb, or empty Lm (both 1 × $10^5$ transferred cells) 1 day later. Expression of activation marker CD25 and dilution of the proliferation dye in donor cells was examined on day 5 post-infection. Representative mice out of seven in total from three independent experiments are shown. The cells from the Lm-OVA infected mice are the same as used for the analysis in Fig 5G.


© 2018 The Authors
