# Supplementary material for: Strong homeostatic TCR signals induce formation of self‐tolerant virtual memory CD8 T cells
Source: EMBO J. 2018 May 11;37(14):e98518. doi: 10.15252/embj.201798518 (PMC6043851; doi:10.15252/embj.201798518)
Supplement: Supplementary file 2 — Table EV1 [file EMBJ-37-e98518-s002.docx]

| **TCR clone** | **V-gene** | **J-gene** | **CDR3-junction** | **Clone count (naïve/VM)** | | | |
| --- | --- | --- | --- | --- | --- | --- | --- |
|  |  |  |  | **Exp1** | **Exp2** | **Exp3** | **Exp4** |
| **TRAV14 Clones** | | | | **(19/18)** | **(16/16)** | **(21/17)** | **(17/17)** |
| V14-c1 | TRAV14-1 a)  TRAV14D-1  TRAV14D-2 | TRAJ6 | CAAGGNYKPTF | -/9 | -/10 | 12/12 | 1/8 |
| V14-c2 | TRAV14-1 b)  TRAV14D-1 | TRAJ6 | CASGGNYKPTF | 2/6 | -/3 | 4/5 | 2/1 |
| V14-c3 | TRAV14-1 | TRAJ6 | CASGGNYQPIC | -/1 | - | - | - |
| V14-c4 | TRAV14-1 | TRAJ6 | CASGGNYKPTC | -/1 | - | - | - |
| V14-c5 | TRAV14-3 | TRAJ5 | CAASPQVVGQLTF | -/1 | - | - | - |
| V14-c6 | TRAV14-1 c)  TRAV14D-3/DV8 | TRAJ6 | CAAGNYAQGLTF | 2/- | 7/- | 4/- | - |
| V14-c7 | TRAV14-1 | TRAJ31 | CAAGDNNRIFF | 4/- | - | - | 3/- |
| V14-c8 | TRAV14-1 | TRAJ34 | CAAGDTNKVVF | 6/- | - | - | - |
| V14-c9 | TRAV14-1 | TRAJ47 | CAASDPNKMIF | 3/- | - | - | - |
| V14-c10 | TRAV14-1 | TRAJ33 | CAASDNYQFIC | 1/- | - | - | - |
| V14-c11 | TRAV14-1 | TRAJ31 | CAAADNNRIFF | - | -/1 | - | - |
| V14-c12 | TRAV14-1 d) | TRAJ6 | CAGGGNYKPTF | - | -/2 | - | - |
| V14-c13 | TRAV14-1 | TRAJ7 | CAASDNNRLTL | - | 2/- | 1/- | - |
| V14-c14 | TRAV14-3 | TRAJ43 | CAASDNNNNAPRF | - | 1/- | - | - |
| V14-c15 | TRAV14-2 | TRAJ35 | CAARRGFASALTF | - | 3/- | - | - |
| V14-c16 | TRAV14-1 | TRAJ33 | CAAASNYQLIW | - | 1/- | - | - |
| V14-c17 | TRAV14-1 | TRAJ31 | CAASDDNRIFF | - | 2/- | - | - |
| V14-c18 | TRAV14D-3/DV8 | TRAJ39 | CAARDNAGAKLTF | 1/- | - | - | - |
| V14-c19 | TRAV14-1 | TRAJ45 | CAASAAGADRLTF | - | - | - | 1/- |
| V14-c20 | TRAV14-1 | TRAJ6 | CAASETSGGNYKPTF | - | - | - | 1/- |
| V14-c21 | TRAV14-2 | TRAJ33 | CAASGDSNYQLIW | - | - | - | 2/- |
| V14-c22 | TRAV14D-1 | TRAJ6 | CAVGGNYKPTF | - | - | - | 1/- |
| V14-c23 | TRAV14-1 | TRAJ12 | CAASEGGGYKVVF | - | - | - | 1/- |
| V14-c24 | TRAV14-1 | TRAJ31 | CAASDNNRIFF | - | - | - | 2/- |
| V14-c25 | TRAV14-1 | TRAJ7 | CAASDINRLTL | - | - | - | 1/- |
| V14-c26 | TRAV14D-1 | TRAJ49 | CAASSTGYQNFYF | - | - | - | 1/- |
| V14-c27 | TRAV14-1 | TRAJ7 | CAAGDNNRLTL | - | - | - | 1/- |
| V14-c28 | TRAV14-1 | TRAJ6 | CAAGGNYKPIF | - | - | - | -/1 |
| V14-c29 | TRAV14D-3/DV8 | TRAJ52 | CAASADTGANTGKLTF | - | - | - | -/2 |
| V14-c30 | TRAV14-2 | TRAJ12 | CAAWTGGYKVVF | - | - | - | -/1 |
| V14-c31 | TRAV14-1 | TRAJ27 | CAASDNTNTGKLTF | - | - | - | -/1 |
| V14-c32 | TRAV14-1 | TRAJ6 | CAAAGNYKPTF | - | - | - | -/1 |
| V14-c33 | TRAV14-1 | TRAJ58 | CAASAAGTGSKLSF | - | - | - | -/1 |
| **TRAV12 Clones** | | | | **(17/18)** | **(20/12)** | **(18/16)** | **(20/15)** |
| V12-c1 | TRAV12N-3 | TRAJ28 | CALSVRLPGTGSNRLTF | -/2 | - | - | - |
| V12-c2 | TRAV12D-1 | TRAJ23 | CALSAEMNYNQGKLIF | -/2 | - | - | - |
| V12-c3 | TRAV12D-2 | TRAJ27 | CALSDRGTNTGKLTF | -/2 | - | - | -/1 |
| V12-c4 | TRAV12N-3 | TRAJ31 | CALSGSNNRIFF | -/3 | 1/- | 3/- | - |
| V12-c5 | TRAV12N-3 | TRAJ34 | CAPTSNTNKVVF | -/1 | - | - | - |
| V12-c6 | TRAV12N-3 | TRAJ28 | CAPSIKMLTF | -/1 | - | - | - |
| V12-c7 | TRAV12N-3 | TRAJ5 | CALGTQVVGQLTF | -/1 | - | - | - |
| V12-c8 | TRAV12N-3 | TRAJ28 | CAPGSNRLTF | -/3 | 1/- | - | - |
| V12-c9 | TRAV12-3 | TRAJ28 | CALSETGTGSNRLTF | -/2 | 8/- | - | - |
| V12-c10 | TRAV12D-2 | TRAJ4 | CALMLSGSFNKLTF | -/1 | 1/12 | -/8 | - |
| V12-c11 | TRAV12D-2 | TRAJ34 | TNKVVF | 3/- | - | 7/- | 17/1 |
| V12-c12 | TRAV12-3 | TRAJ27 | CALSDQGTNTGKLTF | 2/- | - | 3/- | - |
| V12-c13 | TRAV12-3 | TRAJ32 | CALGMNYGSSGNKLIF | 2/- | - | - | - |
| V12-c14 | TRAV12-3 | TRAJ52 | CALSGGCGANTGKLTF | 1/- | 1/- | - | - |
| V12-c15 | TRAV12N-3 | TRAJ38 | CALSRVGDYCKLIW | 1/- | - | - | - |
| V12-c16 | TRAV12-3 | TRAJ31 | CAPNSNNRIFF | 1/- | - | - | - |
| V12-c17 | TRAV12-3 | TRAJ42 | CALKGGSNAKLTF | 1/- | - | - | - |
| V12-c18 | TRAV12-3 | TRAJ45 | CAPLHTEGADRLTF | 2/- | - | - | - |
| V12-c19 | TRAV12N-3 | TRAJ23 | CALTGEMNYNQGKLIF | 2/- | - | - | - |
| V12-c20 | TRAV12-3 | TRAJ50 | CALSGPASSFSKLVF | 1/- | - | - | - |
| V12-c21 | TRAV12-3 | TRAJ49 | CALSPNTGYQNFYF | 1/- | - | - | - |
| V12-c22 | TRAV12-3 | TRAJ27 | CALSVPNTNTGKLTF | - | 1/- | - | - |
| V12-c23 | TRAV12-3 | TRAJ5 | CAPGTQVVGQLTF | - | 1/- | - | - |
| V12-c24 | TRAV12D-1 | TRAJ32 | CALSDGSSGNKLIF | - | 2/- | - | - |
| V12-c25 | TRAV12-3 | TRAJ2 | CALSVMNTGGLSGKLTF | - | 1/- | 1/- | - |
| V12-c26 | TRAV12D-2 | TRAJ4 | CALSQESGSFNKLTF | - | 1/- | - | - |
| V12-c27 | TRAV12D-1 | TRAJ42 | CGLGGGSNAKLTF | - | 1/- | - | - |
| V12-c28 | TRAV12D-2 | TRAJ25 | CALSGVPGANTGKLTF | - | 1/- | - | - |
| V12-c29 | TRAV12D-2 | TRAJ37 | CALSDRRTGNTGKLIF | - | - | 1/- | - |
| V12-c30 | TRAV12N-3 | TRAJ38 | CALSRVGDNSKLIW | - | - | 3/8 | - |
| V12-c31 | TRAV12-3 | TRAJ27 | CALSDIGTNTGKLTF | - | - | - | 1/- |
| V12-c32 | TRAV12D-2 | TRAJ26 | CALSDAAQGLTF | - | - | - | 1/- |
| V12-c33 | TRAV12D-25 | TRAJ30 | CALSADDTNAYKVIF | - | - | - | 1/- |
| V12-c34 | TRAV12D-2 | TRAJ33 | CALSDHNSNYQLIW | - | - | - | -/1 |
| V12-c35 | TRAV12D-2 | TRAJ30 | CALSSDTNAHKVIF | - | - | - | -/1 |
| V12-c36 | TRAV12D-2 | TRAJ43 | CALSGNNAPRF | - | - | - | -/2 |
| V12-c37 | TRAV12-3 | TRAJ32 | CALSDPYGSSGNKLIF | - | - | - | -/1 |
| V12-c38 | TRAV12N-3 | TRAJ15 | CAPYQGGRALIF | - | - | - | -/1 |
| V12-c39 | TRAV12N-3 e) | TRAJ23 | CALSDRNYNQGKLIF | - | - | - | -/2 |
| V12-c40 | TRAV12-3 | TRAJ42 | CALGSNAKLTF | - | - | - | -/1 |
| V12-c41 | TRAV12-3 | TRAJ42 | CALRTQVVGQLTF | - | - | - | -/1 |
| V12-c42 | TRAV12-3 | TRAJ27 | CALSDRHTNTGKLTF | - | - | - | -/1 |
| V12-c43 | TRAV12N-3 | TRAJ39 | CALSDRYAGVILTF | - | - | - | -/1 |
| V12-c44 | TRAV12N-3 | TRAJ34 | CALSELSSNTNKVVF | - | - | - | -/1 |
